# CeO_2_ Structure Adjustment by H_2_O *via* the Microwave–Ultrasonic Method and Its Application in Imine Catalysis

**DOI:** 10.3389/fchem.2022.916092

**Published:** 2022-05-31

**Authors:** Xijiang Chang, Huihui Ding, Jingxia Yang

**Affiliations:** ^1^ College of Science, Donghua University, Shanghai, China; ^2^ College of Chemistry and Chemical Engineering, Shanghai University of Engineering Science, Shanghai, China

**Keywords:** CeO_2_, catalyst, imine synthesis, microwave synthesis, microwave-ultrasonic combined method

## Abstract

CeO_2_ with fusiform structures were prepared by the combined microwave–ultrasonic method, and their morphologies and surface structure were changed by simply adding different amounts of H_2_O (1–5 ml) to the precursor system. The addition of H_2_O changed the PVP micelle structure and the surface state, resulting in CeO_2_ with a different specific surface area (64–111 m^2^ g^−1^) and Ce^3+^ defects (16.5%–28.1%). The sample with 2 ml H_2_O exhibited a high surface area (111.3 m^2^∙g^−1^) and relatively more surface defects (Ce^3+^%: 28.1%), resulting in excellent catalytic activity (4.34 mmol g^−1^ h^−1^).

## Highlights

1. Fusiform-like CeO_2_ was prepared by the combined microwave–ultrasonic method.
2. 1–5 ml H_2_O in the precursor can influence the morphology, surface area, and Ce^3+^% of the CeO_2_ catalyst.
3. H_2_O changed the PVP micelle structure, leading to modulation of the CeO_2_ surface state.
4. For imine synthesis, CeO_2_ with 2 ml H_2_O showed 2-times higher activity than the control without H_2_O.

## Introduction

Imine compounds are important intermediates for many biological, agricultural, and pharmaceutical compounds, such as alkaloids, membered heterocycles, and nitrogen heterocycles (Martin, 2009; Kaldas et al., 2017); thus, it is of great importance to develop a new synthesis approach for imine. CeO_2_ is a new promising catalyst for imine synthesis, which can overcome the problems caused by traditional acid/base catalysts (Tamura and Tomishige, 2015) and bring a lot of advantages, such as mild synthesis conditions and fast separation. Many research studies have focused on CeO_2_ application in imine synthesis (Geng et al., 2016; Long et al., 2019a; Long et al., 2019b; Cao et al., 2020; Rizzuti et al., 2020; Tamura and Tomishige, 2020), and it has been reported that CeO_2_ morphologies can greatly influence the catalytic activity for imine synthesis (Zhang et al., 2017; Yang et al., 2018; Zhang et al., 2018; Yang et al., 2020a; Yang et al., 2020b), which have a close relationship with the CeO_2_ specific surface area and Ce^3+^ defects.

The preparation approach can effectively change the structure of CeO_2_, especially using the microwave method. Microwaves can heat the target solution by interacting with the solution molecules; thus, the solution can be heated fast and uniformly, which can effectively improve the product quality (Reddy et al., 2012; Li et al., 2021; Ma et al., 2021). Besides, other energies, such as UV-light and ultrasonic force, can work together with microwaves to achieve a new CeO_2_ material structure *via* the Multifunctional Microwave Synthesis and Extraction Workstation (Yang et al., 2020a). Ultrasonic force is a kind of energy-accumulated mechanical vibration waves with thermal effect, mechanical effect, and cavitation effect (Thompson and Doraiswamy, 2000; Dalas, 2001; Shen, 2009), which can be used to accelerate the reaction rate and improve particle dispersion during material synthesis.

Microwave- or ultrasonic-assisted technology for CeO_2_ synthesis has developed very rapidly in recent years (Leonelli and Mason, 2010; Phuruangrat et al., 2017; Zhao et al., 2018; Mousavi-Kamazani and Ashrafi, 2020; Chen et al., 2021; Zhai et al., 2021), and it has been found that different CeO_2_ structures were obtained by using different energy inputs with the same solution (Yang et al., 2020a). Applying microwaves and ultrasonic waves together to the same solution can synthesize materials with a novel structure. In this way, the heat and mass can be transferred much better, and a new material structure can be achieved by changing the solution very slightly.

In this work, a series of CeO_2_ nanomaterials was synthesized by a combined microwave–ultrasonic method. Different CeO_2_ structures can be achieved only by changing the amount of deionized water in the solvent. The obtained CeO_2_ exhibited different catalytic activities for imine synthesis, and their structures were further characterized by XRD, IR, SEM, XPS, and BET to figure out the materials’ structure and the reason behind it.

## Experimental

### Synthesis of CeO_2_

**FIGURE 1:**
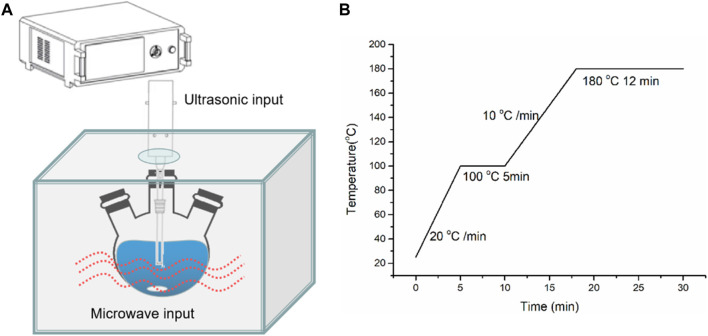
Sketch of combined microwave–ultrasonic synthesis **(A)** and heating program **(B)**.

All chemicals were provided by Adamas Reagents and used as received. The combined microwave-ultrasonic method was used to synthesis CeO_2_, using Ce(NO_3_)_3_·6H_2_O as the starting precursor, PVP as the structure directing agent, and acetic acid (HAC) as the mineralizer. The solvents were formed by ethylene glycol (EG) and DI-H_2_O, with a fixed total volume of 35.0 ml. The synthesis procedures were all the same; only the solvent content was changed by using different amounts of H_2_O, as shown in Table 1. The typical synthesis procedure was described using CeO_2_-H2 as an example. Ce(NO_3_)_3_·6H_2_O (2.17 g) was dissolved into the mixed solvent (33 ml EG + 2 ml DI-H_2_O) in a three-neck flask; then PVP (1.00 g) was added, and the resultant solution was stirred for 2 h. Finally, HAC (2.0 ml) was dropwise added, and the solution was further stirred for 15 min. Then the three-neck flask was transferred to a Multifunctional Microwave Synthesis and Extraction Workstation [Uwave-2000 from SINEO Microwave Chemistry Technology (China) Co. Ltd.] using microwave and ultrasonic force as the energy input, as shown in Figure 1A. The heating program is shown in Figure 1B, where the temperature is maintained at 100°C for 5 min and at 180°C for 12 min. After cooling naturally, the solids were centrifuged, washed by ethanol and DI-H_2_O four times, and dried in an oven (80°C) overnight. After that, they were calcined at 500°C (2°C/min) for 1 h in air to remove surface organic residues. In the sample name CeO_2_-H*x*, *x* is the volume of DI-H_2_O added to the solvent.

**TABLE 1:** Reagents used for different sample syntheses.

Sample	Ce(NO_3_)_3_·6H_2_O (g)	PVP (g)	HAC (ml)	EG (ml)	DI-H_2_O (ml)
CeO_2_-H0	2.17	1.00	2.0	35.0	0
CeO_2_-H1	2.17	1.00	2.0	34.0	1.0
CeO_2_-H2	2.17	1.00	2.0	33.0	2.0
CeO_2_-H3	2.17	1.00	2.0	32.0	3.0
CeO_2_-H4	2.17	1.00	2.0	31.0	4.0
CeO_2_-H5	2.17	1.00	2.0	30.0	5.0

### Structure Characterization

The XRD patterns were acquired using a Shimadzu (Japan) D/Max-2500 diffractometer (nickel-filtered CuKα radiation). The morphologies of the CeO_2_ samples were observed using a scanning electron microscope (SEM, Hitachi S-8000, Japan) in the secondary electron scattering mode at 5 kV. N_2_ adsorption/desorption was measured at 77 K by using a Micromeritics ASAP 2020 instrument, and all samples were degassed at 120°C for at least 5 h before testing. X-ray photoelectron spectra (XPS) were recorded using a Thermo Fisher ESCALAB 250 xi (England) using AlKα radiation (1,486.6 eV).

### CeO_2_ as Catalyst for Imine Synthesis

Calcined CeO_2_ (50 mg) was added to a mixture of benzyl alcohol (10 mmoL) and aniline (20 mmoL). The resulting mixtures were stirred vigorously at 500 rpm at 50°C. After a certain time (at least 2 h), the imine product was analyzed using HPLC (Shimadzu LC-20AT, Japan) equipped with a Hypersil ODS C18 column (5 μm in a size of 4.6 mm × 250 mm), and the mobile phase was methanol: water = 8:2. In order to compare with the other reactions, the reaction rates are given as the amount of imine formation per hour per catalyst mass (mmol h^−1^ g^−1^).

## Results and Discussions

**FIGURE 2:**
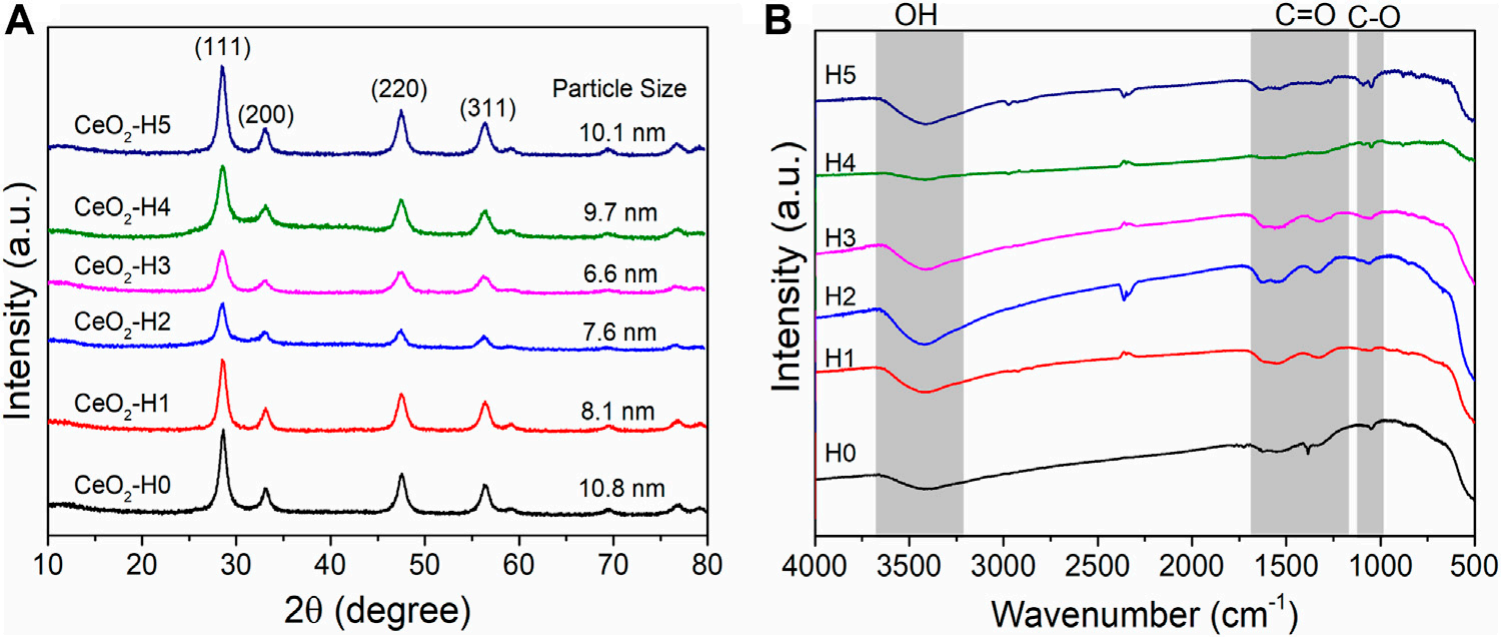
XRD **(A)** and IR **(B)** spectra of CeO_2_ by the combined microwave–ultrasonic method and calcined at 500°C in air for 1 h. The particle sizes in **(A)** are calculated by Scherrer equations based on the strongest peak at 28.5°.

The x-ray diffraction (XRD) patterns of all samples are shown in Figure 2A. Typical CeO_2_ crystalline phases (JCPDF No: 34-0394) appeared for all samples, which were prepared by the combined microwave–ultrasonic method and calcined at 500°C in air for 1 h, similar to the one reported before (Yang et al., 2021), but the crystalline particle sizes were different when calculated by Scherrer equations based on the strongest (111) peak at 28.5°. The crystalline particle size changed in the range of 6.6–10.8 nm, which has a close relationship with the DI-H_2_O amount in the solvent. The CeO_2_-H0 sample, prepared without extra DI-H_2_O, possessed the biggest crystalline particle size of 10.8 nm. By adding extra DI-H_2_O to the precursor solution, the crystalline particle size decreased first (1–3 ml DI-H_2_O) and then increased again (4–5 ml DI-H_2_O). The smallest crystalline particle (6.6 nm) appeared when the extra DI-H_2_O was 3 ml (CeO_2_-H3).

The groups on the particle surfaces were characterized by FT-IR (Figure 2B). After calcination at 500°C for 1 h, most of the organic groups were removed, and only a small amount of C=O (1,300–1,650 cm^−1^) and C-O (1,060 cm^−1^) groups can be observed besides the OH group at c.a. 3,500 cm^−1^ (Guo et al., 2021), indicating a relatively clean surface. These groups may be caused by the adsorption of CO_2_ and H_2_O from the air, which can be further confirmed by XPS results in Figure 5.

**FIGURE 3:**
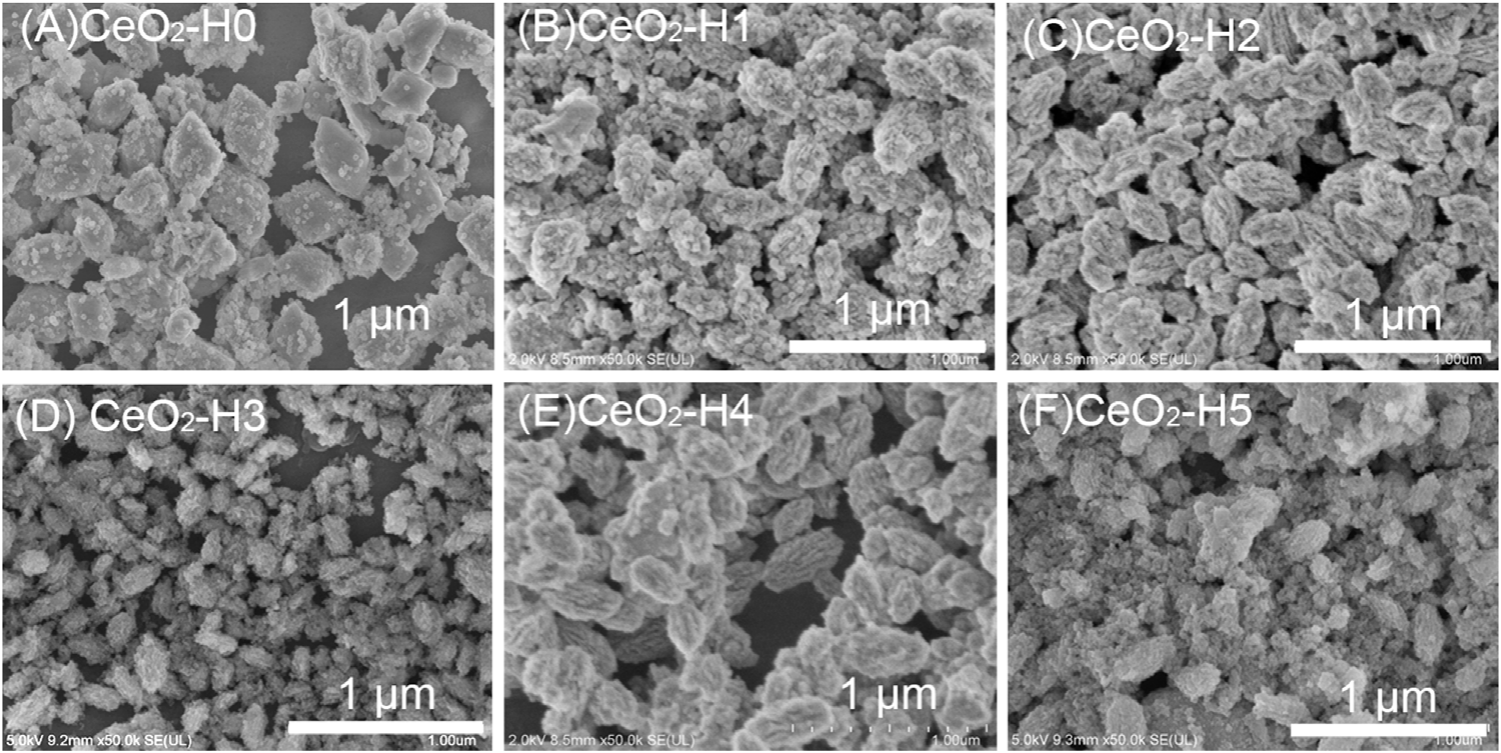
SEM morphologies of CeO_2_ prepared by the combined microwave–ultrasonic method and calcined at 500°C in air for 1 h. **(A)** CeO_2_-H0, **(B)** CeO_2_-H1, **(C)** CeO_2_-H2, **(D)** CeO_2_-H3, **(E)** CeO_2_-H4, **(F)** CeO_2_-H5.

The DI-H_2_O amount in the solvent can influence the morphologies of CeO_2_ particles, as shown in Figure 3. When there was no extra H_2_O in the solution, the CeO_2_-H0 sample (Figure 3A) exhibited an octahedral shape (diameter: 280 nm, length: 460 nm, and aspect ratio: 1.6), formed by aggregation of nanoparticles. The CeO_2_-H1 sample (Figure 3B), with 1 ml extra DI-H_2_O in the solution, also showed a roughly octahedron structure, much more like a shuttle shape (diameter: 180 nm, length: 380 nm, and aspect ratio: 2), and small particles became more obvious in the aggregate. CeO_2_-H2 (Figure 3C) possessed a similar structure to CeO_2_-H1; only the diameter became smaller, which was about 140 nm. When increasing H_2_O to 3 ml, CeO_2_-H3 (Figure 3D) showed a smaller aggregation structure, with a diameter of 80 nm and length of 200 nm (aspect ratio: 2.5). By further increasing H_2_O to 4 ml, the size of the aggregate in CeO_2_-H4 (Figure 3E) increased again, exhibiting a structure similar to that of samples CeO_2_-H1 and CeO_2_-H2. Nevertheless, the octahedron/shuttle shape collapsed for CeO_2_-H5 (Figure 3F); most were small particles, and only few aggregates can be observed. CeO_2_ shuttles were previously synthesized by the hydrothermal method under similar conditions (Guo et al., 2008), but the size (diameter: 800 nm) was much larger than that of the samples in this work.

**FIGURE 4:**
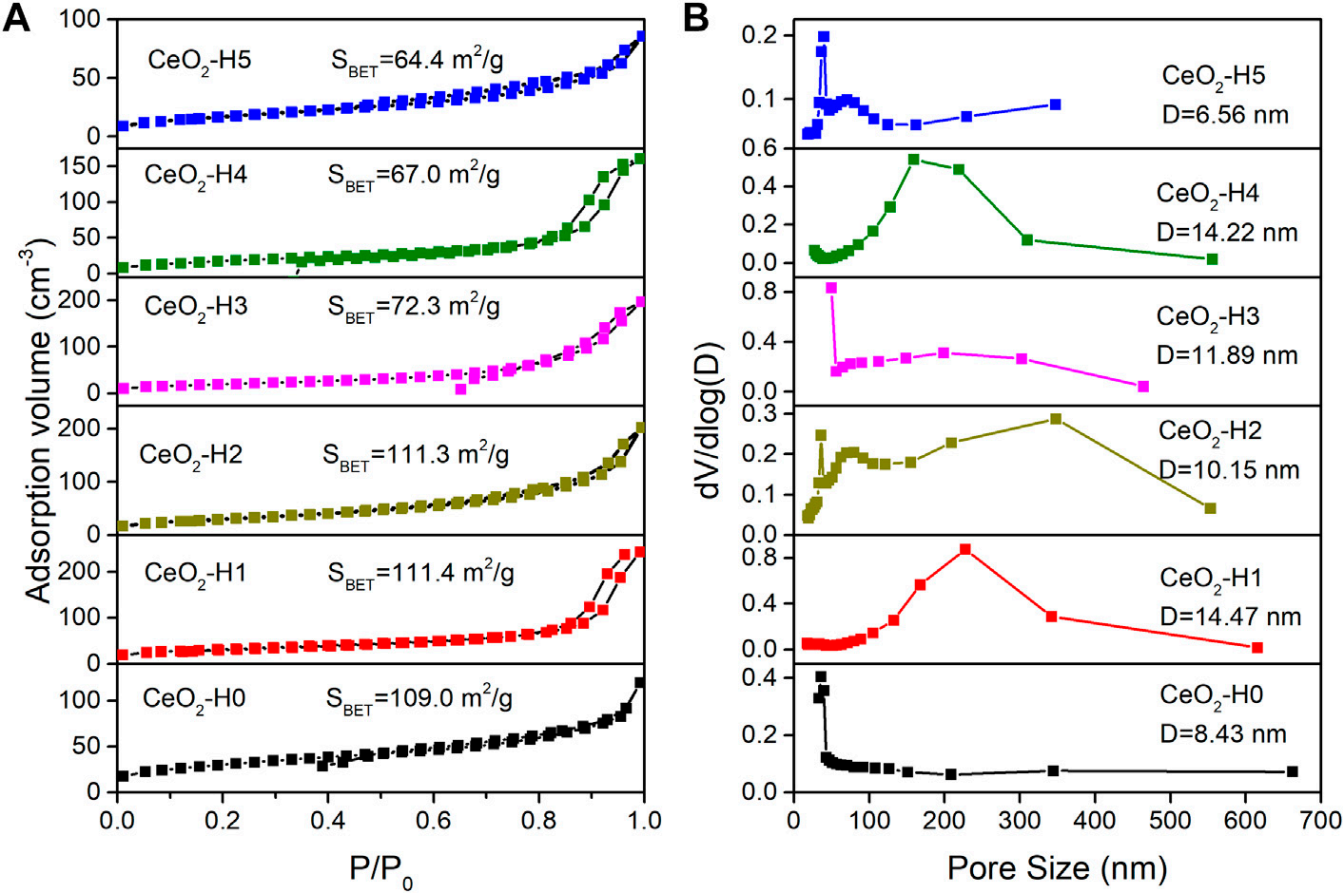
N_2_ sorption isotherm **(A)** and pore size distribution **(B)** of CeO_2_ prepared by the combined microwave–ultrasonic method and calcined at 500°C in air for 1 h.

The N_2_ adsorption–desorption isotherm is used to characterize the materials’ surface area, as shown in Figure 4A, and the pore diameter distribution is shown in Figure 4B. According to the IUPAC classification (Sing et al., 1985; Yang et al., 2020c; Zhang et al., 2021), the hysteresis loop of the N_2_ adsorption isotherm belonged to type VI, indicating that the materials were mesoporous. The specific surface area of CeO_2_-H2 (S_BET_: 111.3 m^2^/g) was similar to that of CeO_2_-H1 (S_BET_: 111.4 m^2^/g), larger than the values of other samples, while the minimum S_BET_ (64.4 m^2^/g) belonged to CeO_2_-H5. Besides the surface area, the H_2_O amount also influenced the pore size distribution, as shown in Figure 4B. When there was no additional H_2_O added (sample CeO_2_-H0), the pore size distribution was relatively narrow, and almost all pore sizes were below 10 nm. When H_2_O was added to the system, the pore size distribution became broader, especially for sample CeO_2_-H2. This confirmed that adding H_2_O can influence the samples’ morphologies. This may be related to the change in the PVP micelle structure. The extra H_2_O addition to the synthesis system changed the arrangement of PVP, resulting in the agglomeration alternation in CeO_2_ particles, and the shape of CeO_2_ became irregular along with H_2_O addition, as shown in Figure 3 and illustrated in Figure 6. The agglomeration variations in CeO_2_ particles presented a different surface area and pore size distribution.

**FIGURE 5:**
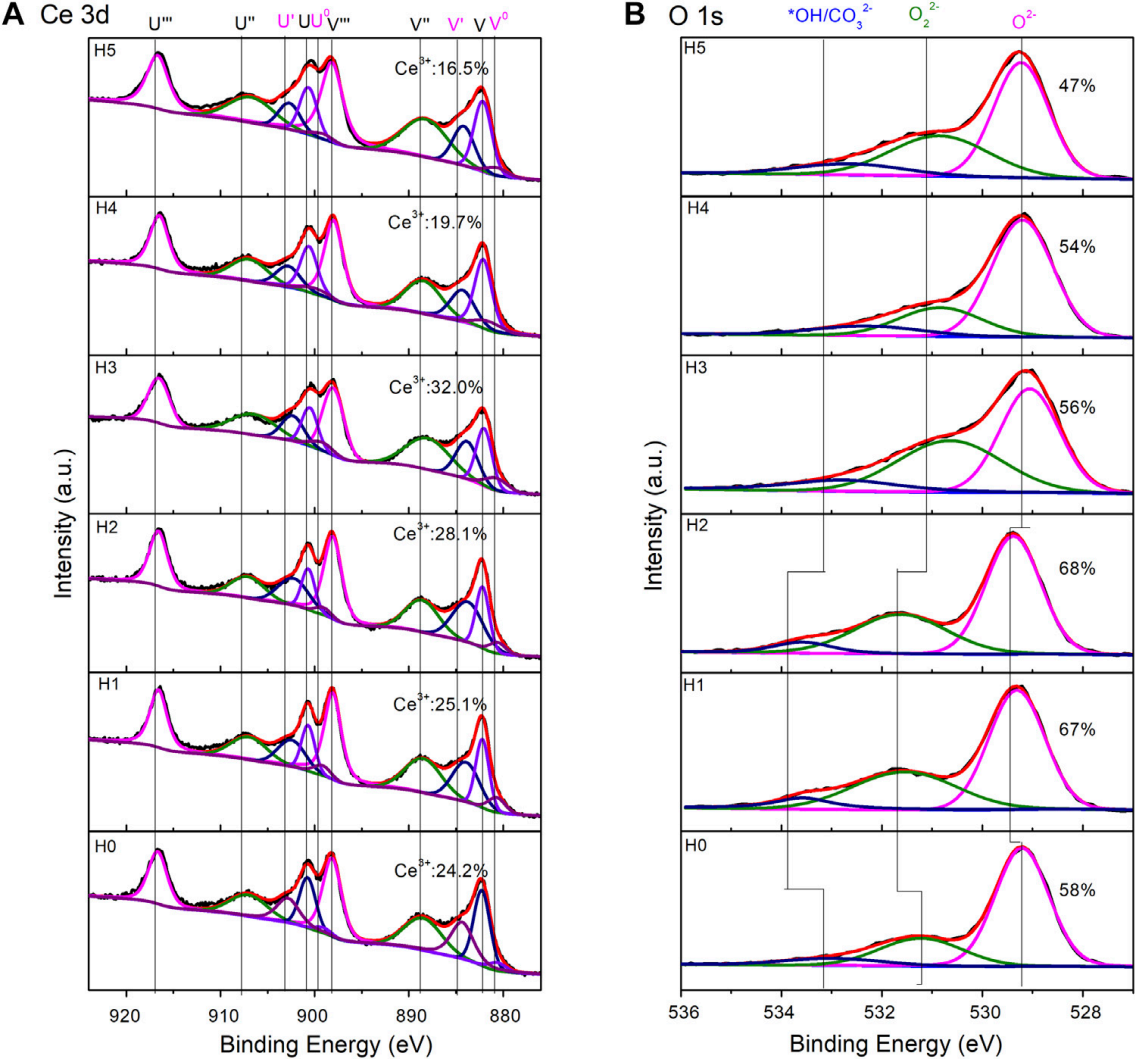
XP spectra of CeO_2_ by the combined microwave–ultrasonic method and calcined at 500°C in air for 1 h. **(A)** is the region of Ce 3d, in which V^0^, V′, U^0^, and U′ belong to Ce^3+^. **(B)** is the region of O 1 s.

**FIGURE 6:**
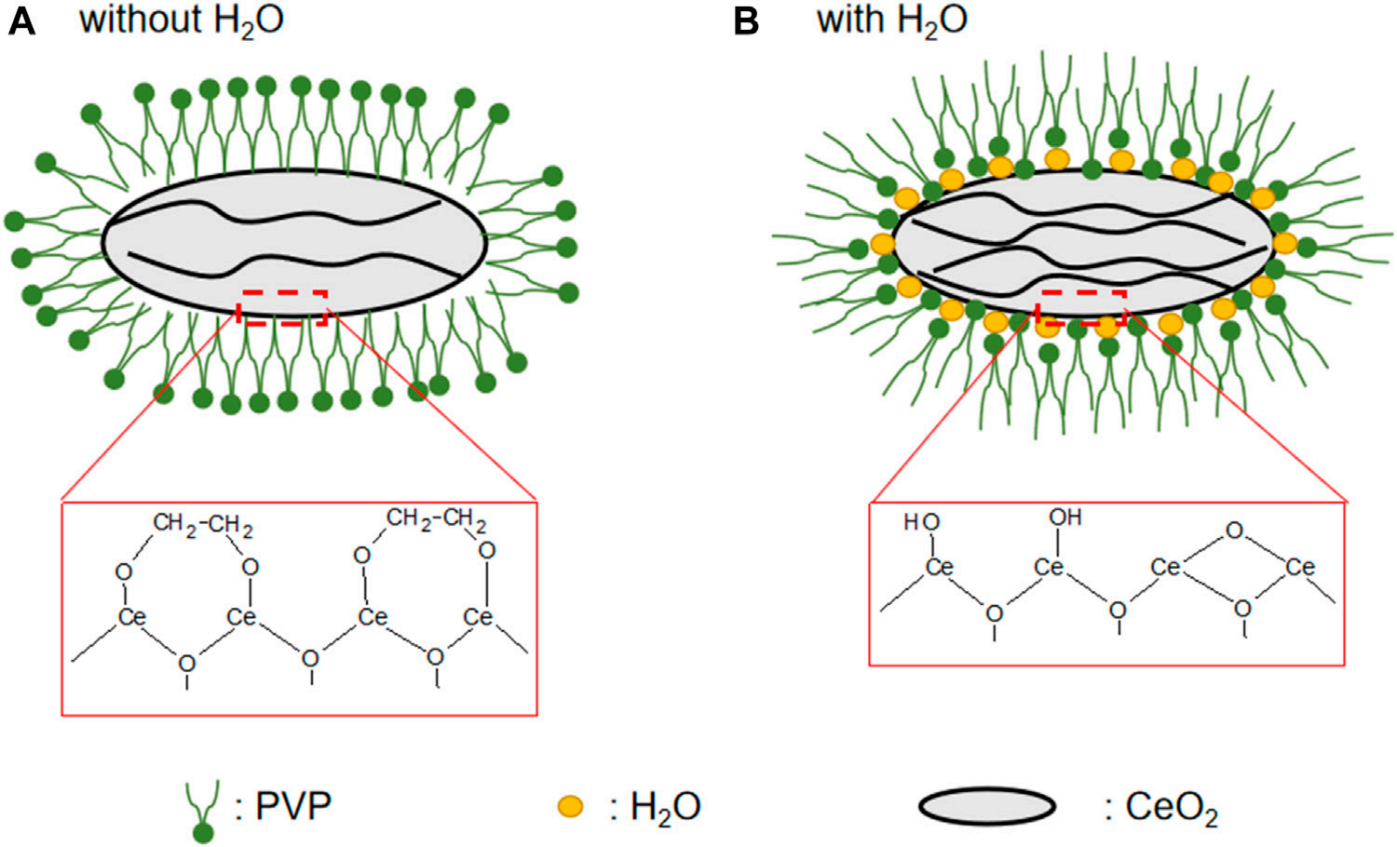
Sketch of the influence of H_2_O on the structure of CeO_2_. **(A)** Without H_2_O. **(B)** With H_2_O.

**FIGURE 7:**
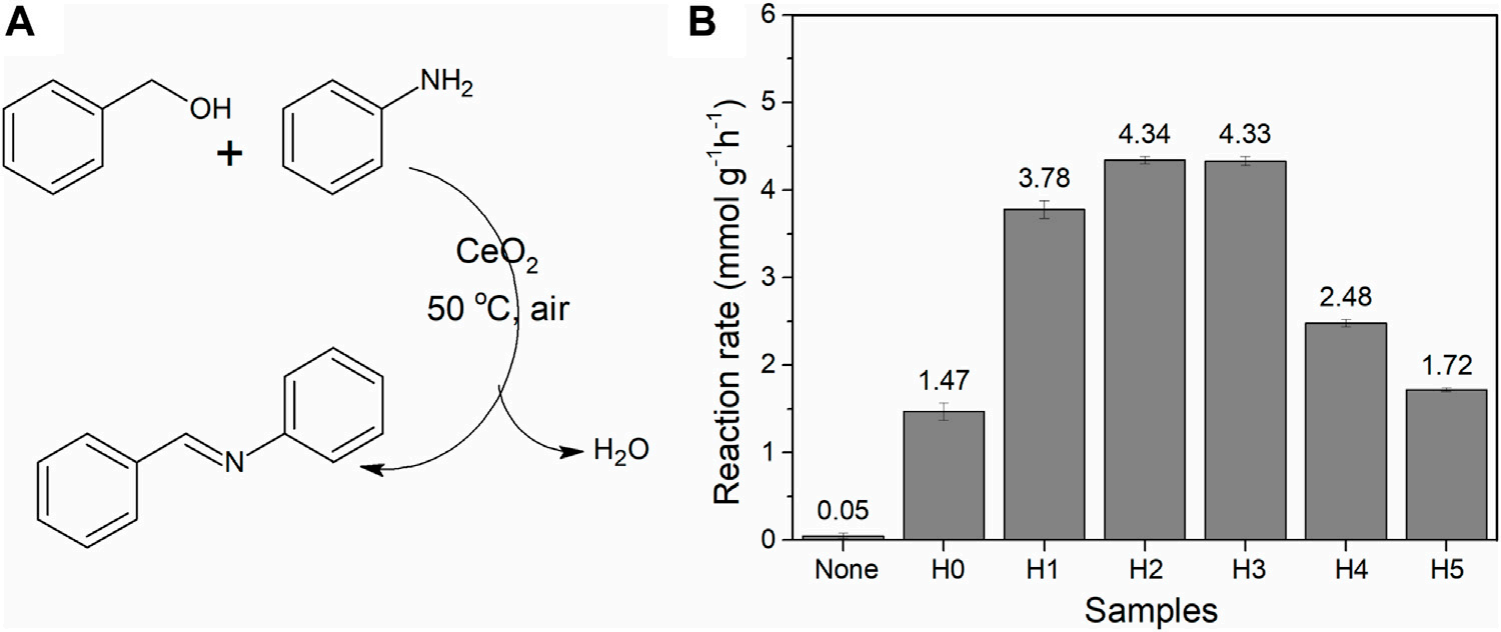
**(A)** Reaction for imine synthesis. **(B)** Imine conversion of CeO_2_ (50 mg) synthesized in different amounts of water (amount of catalyst per hour; all values of conversion are calculated below 20%). The experiment was repeated three times, and the average conversion value was given with an error bar.

XPS is used to characterize the CeO_2_ surface states (Figure 5). Ce^3+^ defects are important and have a close relationship with the catalytic activity of CeO_2_. In the Ce3d region (Figure 5A), Ce^3+^ ratios were calculated using the previous method, by fitting the area of Ce^3+^ and Ce^4+^ peaks (Zhang et al., 2017; Yang et al., 2018; Yang et al., 2020a). The CeO_2_-H0 sample had a Ce^3+^ ratio of 24.2%. The addition of H_2_O increased the Ce^3+^ ratio first and then decreased the number of Ce^3+^ defects. The highest Ce^3+^ ratio (32.0%) belonged to the CeO_2_-H3 sample, with 3 ml H_2_O added to the synthesizing system. CeO_2_-H2 possessed a Ce^3+^ ratio of 28.1%, which was the second top of all Ce^3+^ ratios. Besides, the O1s region can be fitted into three peaks: OH*/CO_3_^2−^, O_2_^2−^, and O^2−^ (Chen et al., 2013). OH*/CO_3_^2−^ and O_2_^2−^ were normally caused by the organic residuals or the surface impurity caused by CO_2_ and H_2_O adsorption, and they can block the active sites of CeO_2_ (Ferreira et al., 2012; Jiang et al., 2015; Yang et al., 2018). Thus, the higher the O^2−^ ratio is, the cleaner the CeO_2_ surface is, indicating more active sites can be exposed. From the O1s region in Figure 5B, it can be seen that the CeO_2_-H2 sample possessed the highest O^2−^ ratio (68%), suggesting that it has the cleanest surface, which is beneficial to the catalytic activity. The highest O^2−^ ratio of CeO_2_-H2 may be because a suitable amount of H_2_O promoted the cleavage of organic residuals, as illustrated in Figure 6. This may compensate the slightly lower Ce^3+^ ratio of the CeO_2_-H2 sample, resulting in the highest catalytic activity for imine synthesis, as shown in Figure 7.

From the above-mentioned characterization, the addition of H_2_O can change the morphology of CeO_2_, which can further alter the specific surface area and the surface Ce^3+^ defects. These changes may be related to the PVP micelle structure change and the CeO_2_ surface coordination state induced by H_2_O, as sketched in Figure 6. When no H_2_O is added to the system, the CeO_2_ surface is covered with EG molecules, and the surface is hydrophobic. Thus, the PVP formed a micelle structure, with the hydrophobic ends toward the CeO_2_ agglomeration particles. When H_2_O is added to the system, it can react with the Ce-precursor, forming a Ce-OH structure; thus, the CeO_2_ surface transformed from hydrophobic to hydrophilic, and the PVP formed a micelle structure, with the hydrophilic ends toward to the CeO_2_ particles. Besides, Ce-OH can also yield a cleaner surface, and the condensation between -OH can happen to yield oxygen vacancies and H_2_O, yielding a higher Ce^3+^ ratio. Meanwhile, H_2_O can also be adsorbed onto the CeO_2_ surface, which can interact with the microwave and sonic energy much better than EG can, causing more wrinkles on the CeO_2_ particles to increase the specific surface area. However, when more than 4 ml of H_2_O was added into the system, the PVP micelle structure was destroyed, and no shuttle structure can be observed.

When synthesized CeO_2_ was used for imine synthesis, they exhibit different activities (Figure 7). The order of imine conversion is CeO_2_-H2 (4.34 mmol g^−1^ h^−1^) ≈ CeO_2_-H3 (4.33 mmol g^−1^ h^−1^) > CeO_2_-H1 (3.78 mmol g^−1^ h^−1^) > CeO_2_-H4 (2.48 mmol g^−1^ h^−1^) > CeO_2_-H5 (1.72 mmol g^−1^ h^−1^) > CeO_2_-H0 (1.47 mmol g^−1^ h^−1^). The reaction rates of all the samples were far better than the 0.46 mmol g^−1^ h^−1^ reported in Angew (Tamura and Tomishige, 2015). It has been reported earlier that CeO_2_ can be used as an easily separable catalyst for imine synthesis at low temperature (<100°C) (Zhang et al., 2017; Zhang et al., 2018; Chen et al., 2020; Wu et al., 2021) and oxygen vacancies (Ce^3+^ defects) played an important role during this reaction. The Ce^3+^ vacancies can promote the transition of benzyl alcohol to benzaldehyde, and the latter can easily couple with aniline to form an imine compound (Tamura and Tomishige, 2015; Zhang et al., 2017; Qin et al., 2019). From the above-mentioned results, it can be seen that CeO_2_-H2 possessed the largest surface area (111.4 m^2^/g) and a relatively high Ce^3+^ ratio (28.1%), which contributed to the highest imine reaction rate. CeO_2_-H3 exhibited a similar catalytic activity, which may be due to its highest Ce^3+^ ratio (32.0%), but its surface area (72.3 m^2^/g) is a little bit lower than that of CeO_2_-H2. The results confirmed that both surface area and Ce^3+^ ratio are important to the CeO_2_ catalytic properties for imine synthesis.

## Conclusion

Different sizes of fusiform CeO_2_ were synthesized by the combined microwave–ultrasonic method, and H_2_O was added to change the structure of CeO_2_. By changing the amount of H_2_O from 1 to 5 ml, the specific surface area of CeO_2_ changed from 111 m^2^/g to 64 m^2^/g, while the Ce^3+^ ratio varied in the range of 16.5% and 32.0%. The sample with 2 ml H_2_O has the largest surface area (111.4 m^2^/g) and a relatively high Ce^3+^ ratio (28.1%), indicating that the CeO_2_-H2 sample has the largest amount of exposed surface oxygen vacancies, and the imine reaction rate is 4.34 mmol g^−1^ h^−1^, which is almost three times the rate (1.47 mmol g^−1^ h^−1^) produced by the sample without addition of water.

## Data Availability

The original contributions presented in the study are included in the article/Supplementary Material; further inquiries can be directed to the corresponding author.
